# Kimchi throughout millennia: a narrative review on the early and modern history of kimchi

**DOI:** 10.1186/s42779-023-00171-w

**Published:** 2023-04-03

**Authors:** Reggie Surya, David Nugroho

**Affiliations:** 1 Food Technology Department, Faculty of Engineering, Bina Nusantara University, Jakarta, 11480 Indonesia; 2 Department of Integrated Science, Faculty of Science, Khon Kaen University, Khon Kaen, 40002 Thailand

**Keywords:** Kimchi, Kimchi war, History, Korea, Ethnic food

## Abstract

Kimchi, the traditional fermented vegetable dish from Korea, has been an integral part of the Korean food culture for thousands of years. Today, it is considered as a symbol of identity for Korean people and is globally appraised as a healthy food. The international standard of kimchi was stipulated by the Codex Alimentarius Commission in 2001, and kimjang, the traditional Korean communal activity of making kimchi, was listed as a UNESCO’s Intangible Cultural Heritage of Humanity in 2013. The international recognition that kimchi has today was not obtained easily since there have been international disputes with regard to its standard and originality. Many historical events have shaped the face of kimchi as it is today. Thus, it appears primordial to understand the hallmark historical events defining the development of kimchi from a traditional food consumed by only Koreans in the past to a renowned global healthy food today. This review explores the historical values of kimchi by focusing on both its early and modern history. The early history of kimchi gathered from different classical literature works suggests the existence of kimchi in Korea since thousands of years ago. The modern history of kimchi highlights different events, including the globalization and commercialization of kimchi, the “kimchi wars” against neighboring countries and the international branding of kimchi as a healthy food. Furthermore, this review also discusses the polemics of kimchi, particularly in terms of its originality. Understanding the historical values of kimchi would make people see kimchi not only as an ethnic food from Korea, but also as a valuable global heritage for the world that needs preserving.

## Introduction

Food as a basic necessity has been an integral part of human civilization since ancient times. A food culture is shaped and developed through a long process that involves the common attitudes, beliefs and practices unique to a community. Therefore, certain ethnic foods are strongly associated with the identity and culture of a specific community. Through time, food evolved along with the advancement of human civilization, built a sense of belonging in society and finally become an integral part of the culture commonly accepted in the society [1]. Today, consuming ethnic foods is still a common practice in many countries, including Korea with its globally recognized dish, kimchi.

**Fig. 1:**
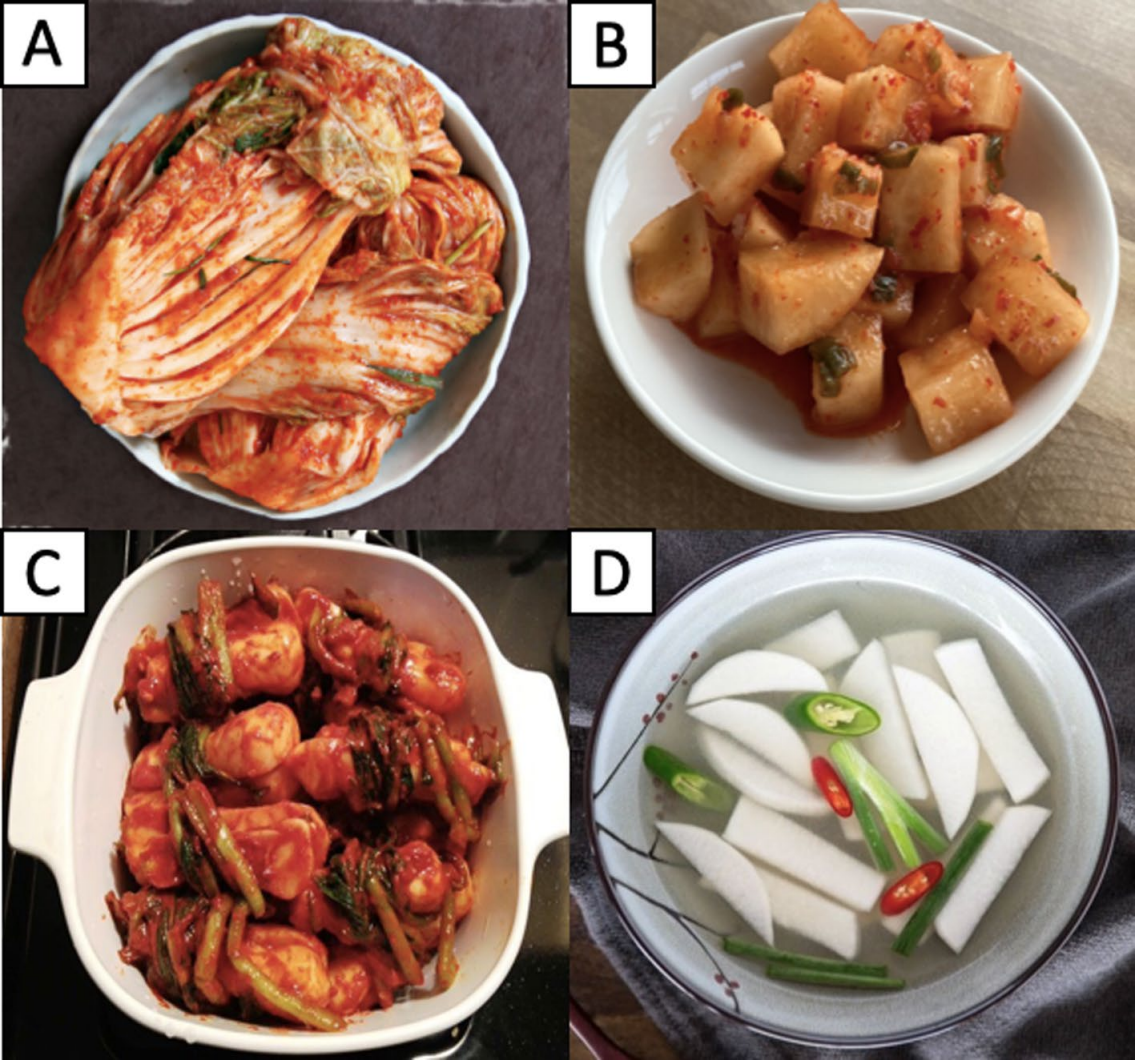
Different famous variations of kimchi in Korea, including **A** *baechu* kimchi made from napa cabbage, **B** *kkakdugi* kimchi made from Korean radish, **C** *chonggak* kimchi made from ponytail radish and **D** *dongchimi* kimchi, a type of watery (*mul*) kimchi usually consumed as soup in winter

Kimchi is a broad term used to define lactic acid-fermented vegetable dish originating from Korea. There are currently more than 200 variations of kimchi in Korea [2], among which *baechu* kimchi (Fig. 1A) made from napa cabbage (*Brassica rapa* subsp. *pekinensis*) is the most well-known and often addressed as simply kimchi [2]. *Baechu* kimchi is the most consumed type of kimchi in Korea, followed by *kkakdugi* kimchi (Fig. 1B) made from Korean radish and *chonggak* kimchi (Fig. 1C) made from ponytail radish [3]. Some other variations of kimchi include green onion (*pa*) kimchi, mustard leaf (*gat*) kimchi, perilla leaf (*kkaenip*) kimchi and cucumber (*oi sobagi*) kimchi [4]. Some kimchi variations are categorized as watery (*mul*) kimchi usually consumed as soup, including *dongchimi* kimchi (Fig. 1D) and *nabak* kimchi [4]. Kimchi is made by fermenting vegetables and additional ingredients (seasonings) in a closed container preferably at a low temperature to allow slow microbial activity and flavor development, as well as long preservation [5]. Some common seasonings used in kimchi making are garlic, ginger, radish, carrot, green onion, fermented seafood (*jeotgal*) and red chili powder (*gochugaru*) [5]. The presence of the latter brings uniqueness to kimchi from other ethnic fermented vegetables, such as *pao cai*, *tsukemono* and *sauerkraut* [6]. The fermentation of kimchi takes place due to the activity of lactic acid bacteria (LAB) producing a plethora of organic acids and other compounds that contribute to the unique and complex flavor of kimchi [7].

Kimchi is consumed daily by Korean people and has been an integral part of Korean food culture for millennia [6]. Indeed, kimchi is rich in historical and philosophical values that reflect the Korean way of life through different times [4]. As previously stated, kimchi grew and walked along with Korean people through times. As Korea entered the industrialization era in the 1960s, it constantly experienced a rapid economic growth until today [8]. With Korea being one of the opulent and economically strong countries in the world, kimchi also gained attention from international audience. Kimchi experienced a radical change in the past 40 years, from being practically unseen and considered as an inferior food before the 1980s to becoming a globally renowned health food today [9].

As kimchi started to obtain global attention and be recognized as a Korean food, it faced many challenges, particularly with regard to its originality and history. There were disputes with the neighboring countries such as China and Japan regarding the standards and economic interests of kimchi happening in the 2000s known as kimchi wars [10]. In addition, polemics regarding the authenticity of kimchi also took place and weakened the identity of kimchi as an original Korean food [10]. For instance, kimchi has been said to have the same root as the Chinese pickled vegetable dish known as *pao cai* and have only existed for 100 years [6]. Korean food scientists have addressed such polemics through scientific publications focusing on the early history of kimchi obtained from the existing classical literature [6, 11]. Their findings were convincing to prove the early history of kimchi in Korea and its existence for thousands of years.

Despite the interest in understanding the early history of kimchi from classical literature, it appears primordial to gather the important facts in the past 40 years that significantly mark the rise and international recognition of kimchi. Altogether, these facts are categorized as the modern history of kimchi that has strongly shaped the face of kimchi as it is today. It is also important to provide an international publication with reliable and scientific information that can be referred to in order to understand the modern history of kimchi. To our knowledge, there has been no publication on the modern history of kimchi gathering the latest important events regarding kimchi development.

This review aims to explore the historical values of kimchi by focusing on both the early and modern history of kimchi. The analysis of the early history of kimchi was performed on classical literature and aimed to demonstrate the authenticity of kimchi and its existence in Korea since ancient times. The modern history of kimchi was interpreted from different hallmark events related to the development and globalization of kimchi. This review is expected to provide thorough and comprehensive information with regard to the history of kimchi throughout millennia. Understanding the historical values of kimchi would make people see kimchi not only as an ethnic food from Korea, but also as a valuable heritage for the world that needs preserving.

## Methodology

The present study focuses on reviewing past studies on the early history of kimchi using scientific articles from different databases, such as Google Scholar and Scopus. Popular references from reliable online resources were collected and compiled to explain the modern history of kimchi since modern events related to kimchi are rarely found in scientific publications. The data were then compiled according to form a comprehensive history of kimchi divided into two categories: early history of kimchi (before the 1980s, but mostly during the periods of Korean dynasties) and modern history of kimchi (from the 1980s until today).

## Brief history of Korea, Korean food and kimchi

Geographically, the Korean peninsula is isolated from neighboring countries. It is surrounded by oceans (the Yellow Sea and the East Sea) on the east, south, and west, and is isolated by rugged mountains to the north, despite being attached to mainland China. In addition, historically, Hwangha culture based on the Hwangha (黃河) River and Yoha culture based on the east of Yoha (遼河) River differed in terms of agriculture, food and language [12]. Such conditions allowed the ancient Koreans to develop a distinct ethnicity and unique culture from the neighboring countries, including China [13]. Prior to the foundation of the Qing Dynasty, China was led by ethnic groups especially the Han Chinese, during the Three Kingdoms and the Ji, Tang, and Ming Dynasties. During this period, Korea was inhabited by Altaic people (of Mongolian descent) and developed its own language and history [14]. The progression of Korean history passed through different periods, including Gojoseon (~ 2333 BC-108 BC), Three Kingdoms: Goguryeo, Baekje and Silla (57 BC-668 AD), Goryeo (918–1392) and Joseon (1392–1910) Dynasty [14]. Throughout these periods, Korea has always maintained independence from China, which led to Korea’s distinct history and culture [14]. In terms of ethnobiology, Koreans can be distinguished from Chinese from the Mongolian spots that they share with Mongolians [15]. The Korean language (Hangul) is a phonetic language with its own alphabet that belongs to the Altaic language family that differs from the Chinese symbolic language [15]. Therefore, Korean culture developed separately from that of China in both geographic and ethnologic terms. Accordingly, Korea also has its own unique food culture which differs from China [13].

Since ancient times, Korea has had a strong agricultural background implying farming culture with more than 5,000 years of history [16]. This has been proven by the finding of a field from Neolithic Age excavated in Munam-ri, Jukwan-myeon, Goseong-gun, Gangwon-do Province in 2012, the oldest ever found in any East Asian countries including China and Japan [16]. The field with clear plowed rows suggested an advanced farming culture in Korea with grains and vegetables as the main cultivars [17]. The focus on agriculture has shaped the Korean diet to consist of mainly plant-based food [18]. The food culture of Korea developed from the urge to store and preserve food for a longer availability, particularly during the harsh and long winter when many people died of starvation. Unlike in China where pickling and frying were the prevalent methods for food preservation, the limited availability of cooking oils in Korea directed the ancient Koreans to opt for fermentation as a strategy for food preservation. Throughout years of experience, ancient Koreans discovered that salted and seasoned vegetables, beans, seafood and other foodstuff remained edible and even developed a unique flavor after being kept in large earthenware jars (*onggi*) for a certain period of time [13].

The Neolithic Age (8,000 BC) marked the beginning of settled life and agriculture in the Korean Peninsula and its northern part [19]. The gastronomy culture of Korea is suggested to be born during this period. Pottery, one of humanity’s first inventions, is made by combining earth, water and fire. Prior to the invention of pottery, food was stored and transported in containers made from wood, leather, and other organic materials. The invention of pottery enabled people to easily store food and even cook their food, thus marking the beginning of culinary culture. Raised-design pottery (*deonmunui togi*) appearing circa 6000 BC and comb-patterned pottery (*bitsalmunui togi*) appearing approximately a millennium later are among the most important relics proving the gastronomy culture in ancient Korea [19]. This particular example of comb-patterned pottery was excavated from the prehistoric settlement site of Amsa-dong, Seoul [19]. It is a simple V-shaped vessel, with a wide mouth and narrow base, and the entire surface is decorated with engraved lines and dots forming geometric patterns. Both the form and the decorative elements that characterize this pottery are considered unique to Korea and have been rarely found elsewhere outside the Korean peninsula [19]. During the Mumun period (1500 BC), Korean people applied intensive agriculture practices to grow millet, barley, wheat, rice and legumes [20]. Archaeological remains during this period also point to the development of fermented beans [20].

During the Three Kingdoms period, Korea exhibited a rapid cultural evolution. Each kingdom (Goguryeo, Baekje and Silla) developed its own distinct set of cultural practices and foods. For instance, Baekje was known for cold foods such as *namul* [18] and fermented foods like kimchi [21]. The spread of Buddhism and Confucianism in Korea during circa 400 AD slightly influenced the distinct food culture of Korea [21]. Later on, the invasion of the Mongols in the thirteenth century allowed food acculturation in Korea, as demonstrated by the introduction of dumpling (*mandu*), grilled meat, noodles, black pepper and *jeon* (similar to pancake) in Korean cuisine [21].

The culinary culture of Korea exhibited a significant development during the Joseon period [21]. In the *Samguk Sagi* (*History of the Three Kingdoms*), it was stated that Koreans had an advanced fermentation technology [15]. Also in this period, many literature works were written to document agricultural practices and cuisine recipes. The opening of Korea to other countries and the Western world through trading activities brought a further exchange of culture and food. Such activities with China, Japan, the Philippines and European countries have allowed the introduction of foreign crops to Korea to be later incorporated into Korean cuisine, including maize, sweet potatoes, tomatoes, peanuts and squash [21]. In the late Joseon Dynasty, a type of kimchi named *seokbakji* was exclusively created for the noble families from an assortment of ingredients including colorful vegetables, seafood, fermented fish, nuts and sea staghorn. This coveted kimchi is viewed as an early form of *tongbaechu* kimchi made from whole napa cabbage [22] usually prepared during kimjang, the communal preparation of kimchi for winter [4].

## Kimchi as the identity and pride of Korean people

Kimchi is indeed a unique and famous fermented food in Korea, thus renowned as Korea’s representative food. It is the icon of Korea and Koreans take great pride in this culinary culture. Kimchi has become an integral part of Korean food culture for thousands of years. The presence of kimchi is considered as indispensable on Korean table during each meal. On average, a Korean would consume 27.6 g kimchi on a daily basis [23]. The annual per capita kimchi consumption in Korea reached 39.9 kg in 2017 [22]. Kimchi is usually served as a side dish (*banchan*) to be eaten with other elements of a Korean meal (*bapsang*), including steamed rice (*bap*), soup (*guk*), salted dish (*jang*), and other side dishes consisting of vegetables (*namul*) and/or protein dishes (meat and fish) [24]. In Korea, there is a common saying “if you have kimchi and rice, you have a meal” [4]. This expression highlights the important place kimchi has in the Korean food culture. Even without any other dishes, the sole presence of kimchi and rice would suffice to compose a complete Korean meal.

Kimchi as the national food of Korea has been made and eaten by the Koreans even before Taoism and Confucianism, the two major strands of Korean philosophy and religion, were introduced to Korea [25]. Confucianism influenced mainly the ritual food of the Joseon Dynasty [26]. The development of Buddhism as the main religion in Korea only had little impact on Korean food culture. Among ordinary Koreans, with the exception of Buddhist priests, there were very few vegetarians or vegans [27].

The attractive appearance of kimchi as well as its complex flavor development during the slow fermentation process represent the beauty aspects of kimchi [28]. As a healthy food, kimchi represents the basic Korean philosophy of *yak sik dong won*, implying that food and medicine come from the same source or, in other words, food is indeed a medicine [17]. Kimchi also possesses humanistic values that represent the Korean way of life [9], including the dimension of filial piety emphasizing respect toward older people [29]. Some kimchi varieties are prepared from pre-boiled vegetables instead of the raw ones to help the elderly with teeth and digestion problems enjoy kimchi as much as young people do [30].

Kimchi has also been long used as an instrument of gastro diplomacy by the Korean government, particularly to increase the international brand awareness of the nation [31]. In 2010, through the tagline “Taste of Korea,” South Korea introduced Korean food (K-food) as a force for international diplomacy [10]. In the process, the Korean government had to face debates regarding kimchi originality and address some problems including considering the halal aspects of Korean food for promotion in Muslim-majority countries [10, 32]. K-food, including kimchi, is regarded more than a merely culinary product, it is also considered as strategic tools for cultural exchange and economic development. Along with the global popularity of Korean pop (K-pop) and Korean drama (K-drama) initiated in the late 2000s, K-food and kimchi flourished and started to be internationally associated with the identity of Korea as a nation [33]. Korea is a perfect example of how gastro diplomacy can be used as a soft power to build the image of a nation and gain international recognition. Other countries that have succeeded in using gastro diplomacy include Thailand, Japan, Taiwan, Malaysia, Australia, Denmark and Peru [32].

For Korean people, kimchi is a symbol of identity and nationalism [10]. Kimchi is strongly related to the life of the Koreans that it has a deep meaning in the heart of every Korean as a symbol of unity among them. Kimchi is indeed a common element that is known and experienced by every Korean. Through kimchi, Korean people share a common thing that cannot be found elsewhere. This could be the suggested reason behind the love and pride of Korean people for kimchi. According to a poll involving Korean people in 2006, kimchi was cited as the symbol of national culture representing Korea by 22.1% respondents, second only to the national flag of South Korea, *taegeukgi* voted by 34.9% respondents, followed by *Hangul*, the Korean writing system (17.2%), *mugunghwa*, the national emblem-flower (13.9%) and *dogdo*, small islets over which Korea and Japan claimed sovereignty (13.2%) [10].

## Early history of kimchi

Historically, kimchi was born from the intention of Korean people to eat vegetables safely and deliciously later during the cold and harsh winter. They prepared *yangnyeom* (seasonings) firstly by mixing garlic, *gochugaru* (red chili powder), ginger and green onion prior to soaking vegetables in this mixture. Through many years, the ancient Koreans learned techniques of vegetable preservation and found that fermented foods developed a unique and even better flavor [12]. The early existence of kimchi is recorded in classical literature works mostly written in the Chinese language. The Korean alphabet (*Hangul*) only appeared in the documents compiled after the fifteenth century following the official establishment of *Hangul* in 1446 [34]. This chapter discusses the presence of kimchi since ancient times in Korea as shown in different classical literature works. Moreover, the historical evidence of kimjang as an integral element of kimchi in ancient literature is also discussed.

### Kimchi in classical literature

**Fig. 2:**
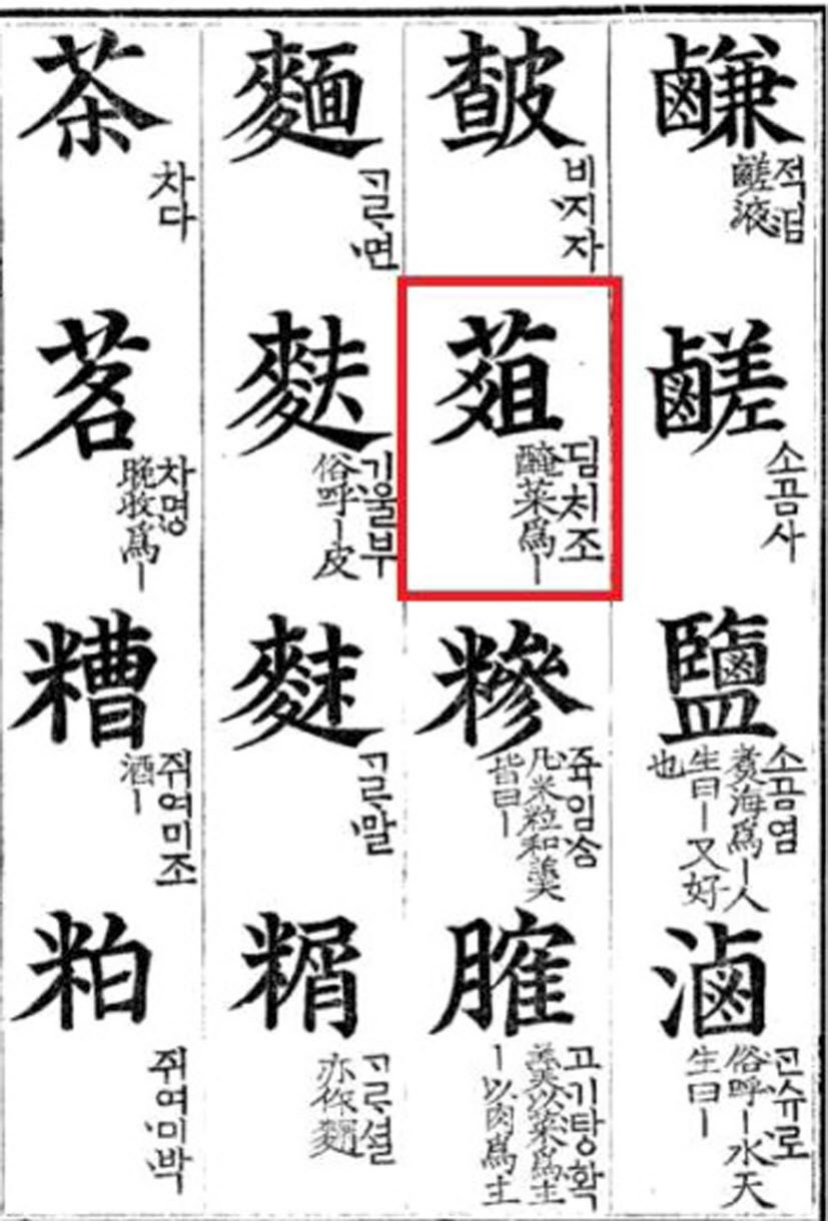
Historical writing showing the word *jeo* (菹) referring to kimchi in the Korean classical literature *Hunmongjahoe* by Choi (1343), a dictionary made for children to look up Chinese characters in Korean after King Sejong created Hangeul (Source: [6])

The oldest classical literature recording the origin of fermented vegetables in Korea is *Sikyung* (the *Classic of Poetry*), a collection of Chinese poetry dating back to the eleventh to seventh centuries BC [6, 11]. In the literature *Hunmongjahoe*, the word *jeo* (菹) appears and refers to a vegetable pickle (Fig. 2). A phrase in the literature says “cucumbers growing on the farm are shredded to make *jeo* and offered to ancestors” [6, 11]. Most Korean kimchi researchers see this sentence as a clue to the appearance and use of early kimchi. The word *jeo* (菹) also differs from the Chinese character used for *pao cai* (泡菜), thus indicating that kimchi and *pao cai* are two distinct foods. Old records did not describe the taste of *jeo* in detail. The *Master Lü’s Spring and Autumn Annals* compiled circa 239 BC states Confucius’ first experience of tasting *jeo*: “hearing that King Wen of Zhou loved *jeo* made of sweet flag, Confucius reluctantly ate it with a wry face; he became used to it after three years.” Based on the descriptor “with a wry face,” researchers postulated that *jeo* was acidic [22]. This indicates that kimchi was not a food familiar to Chinese people, thus suggesting that China would not be the kimchi’s place of origin.

The word *jeo* (菹) also appears in later classical literature, including in the *Samguk Sagi* (*History of the Three Kingdoms*) completed in 1145. This literature is an essential historical record mentioning the existence of kimchi made from Chinese cabbage and Korean red chili pepper (*gochu*) during the Three Kingdoms Period. In this document, there is a description of a cruel way of killing people: “I want to tear a person limb from limb like the way we tear kimchi.” From the description of tearing kimchi, one may conclude that the kimchi referred to is *baechu* kimchi made of cabbage [6, 35]. In a chapter, it is also stated that *gochu* had been existing on the Korean peninsula for over 2,000 years and was widely cultivated during the Three Kingdoms Period [6]. The document contains the word *Chodo*, the island where *gochu* was planted. In addition, the literature also mentions the use of pickle jar (*onggi*) to ferment vegetables, which indicates that fermented vegetables were commonly eaten during the period [6, 11]. Taken together, it is suggested that the cabbage kimchi as we know today has been consumed for about at least 2,000 years in Korea according to *Samguk Sagi*.

Kimchi also appears in artistic literature works, including in a famous poem written by Kyubo Lee in the thirteenth century showing that radish kimchi was a commonplace during the Goryeo period [6]. The word *jiyeom* meaning pickled vegetable is referenced in one of Lee’s poems on the six home garden vegetables entitled *Gapoyukyeong* that states “pickled radish slices make a good summer side-dish / Radish preserved in salt is a winter side dish from start to end / The roots in the earth grow plumper every day / Harvesting after the frost, a slice cut by a knife tastes like a pear” [21]. This is accepted as the first literature mentioning the production of fermented vegetables in Korea. In various other literature works, kimchi has been present under different Korean spellings in Hangul even though it still adopts the same Chinese character, *jeo* (菹). Those names include *yeomje*, *jiyeom*, *janggwa*, *chimchae*, *dimchae* and *yeomchae* [22]. In particular, *chimchae* was the most widely found word, along with *dimchae* referring to kimchi of the Joseon Dynasty [22]. Indeed, a myriad of literature works mentioning kimchi were born during the Joseon period, mainly in the form of agriculture manuals and cookbooks, including the *Suunjapbang* (1540), the *Chubangmun* (circa 1600), the *Yorok* (circa 1600), the *Domundaijak* (1611), the *Sasichanyocho* (1656), the *Eumsikdimibang* (1670), the *Saekgyung* (1676), the *Sanlimkyungje* (1715), the *Cheungbosanlimkyungje* (1766), the *Kyuhapchongsoe* (circa 1800), the *Imwonsipyukji* (1827), the *Dongkuksesiki* (1849) and the *Buinpylji* (1855) [36].

### Kimchi and kimjang culture

**Fig. 3:**
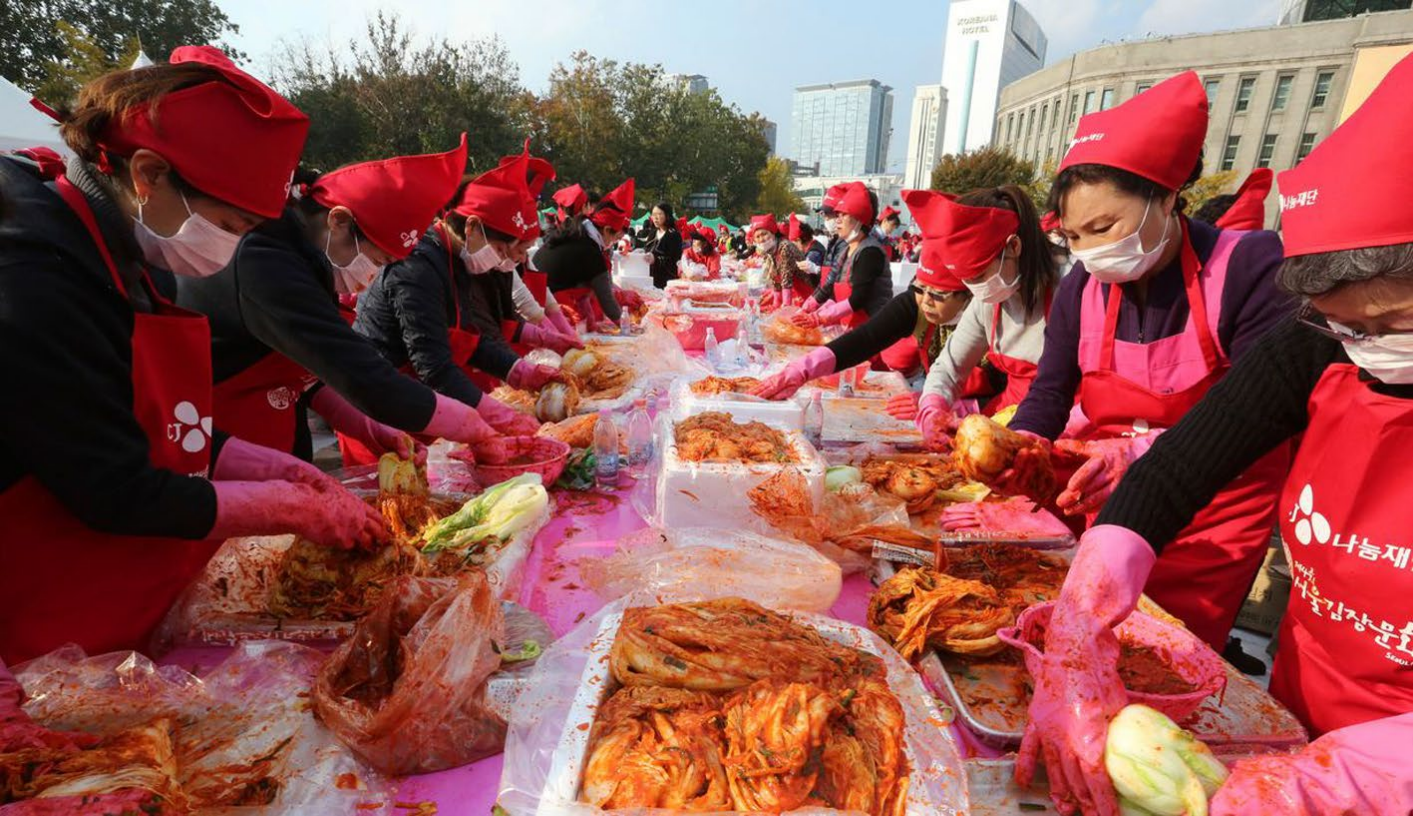
Kimjang, a traditional Korean communal activity of preparing kimchi for consumption in winter that has been registered on the UNESCO’s list of Intangible Cultural Heritage of Humanity since 2013 (Source: [87])

The importance of kimchi in Korean culture is reflected from a special annual event dedicated to the making of kimchi called kimjang (Fig. 3). It is a unique traditional practice of preparing large quantities of kimchi to be consumed throughout winter [4]. Kimjang is a communal activity that involves many participants and the task is shared from a small-scale family level to a large-scale community level. Popularly known as Korea’s winter kimchi party, kimjang is one of the main holidays in Korea and is considered to be the third biggest after *Chuseok* (Korean Thanksgiving) and *Seollal* (Lunar New Year) [37]. Kimjang has been registered on the UNESCO’s list of Intangible Cultural Heritage of Humanity since 2013 [38].

Kimjang is a traditional Korean practice of preparing large quantities of kimchi to consume in winter. It is considered as a unique social experience since the tradition means a lot more than preparing kimchi. Furthermore, it emphasizes the culture of sharing and community spirit, two values anchored in the Korean tradition of kimchi making [39]. Through participation in kimjang, Korean people build a cultural community that transcends social boundaries within society and, thus, allows the real embodiment of the spirit of sharing and promoting solidarity. Kimjang is a beautiful moment of sharing, when people meet and have small talks while performing laborious culinary activities that require a collective hard work. Following kimjang, a big feast usually follows with a communal lunch or dinner amidst conversations, songs and laughter. Furthermore, as an act of generosity, Korean people distribute boxes of kimjang kimchi to their friends and relatives, even to the poor and disadvantaged [39]. Interestingly, kimjang tradition formed when Korea was an agricultural society and still continues even after Korea has developed into a highly industrialized country [40].

The most common kimchi produced during kimjang is *tongbaechu* kimchi made from whole napa cabbage [4]. During kimjang, cabbages are present in large quantities. Female participants are usually responsible for preparing kimjang kimchi while male counterparts perform tasks that require strength, such as carrying ingredients and utensils, digging hole to bury kimchi jars and building a hut over jars. To make kimjang kimchi, napa cabbages are cut in half or quarter prior to salting and marinating with *yangnyeom* (seasonings) [12], including chili powder (*gochugaru*), radish, carrot, green onion, ginger, garlic and fermented seafood (*jeotgal*). These ingredients are collected during all four seasons in Korea throughout a year and represent the philosophy of the five elements, thus making kimjang kimchi a special wholesome Korean delicacy [4]. In traditional kimjang, the chopping and mixing of all ingredients is done manually by hand and often results in muscle aches. All the ingredients are mixed and applied thoroughly into every cabbage leaf before finally the cabbages are wrapped in a certain manner known as *pogi*. In the past, the wrapped cabbages were stored in large clay jars (*onggi*) kept outside the houses or sometimes were buried underground to ensure a slow fermentation process so that kimjang kimchi would be available throughout the winter [13]. A stone jar found in Beopjusa Temple in Chungcheongbuk-do Province in 2000 is believed to be used to keep winter kimchi for 3,000 Buddhist monks in 553 AD [9]. A large kimchi jar believed to date from 640 AD was also found in Jeollado Province [9]. According to a travel essay written by a civil official during the Joseon period named No Jin, there was a legend about a stone jar (similar to the one found at Beopjusa Temple) that was used to make kimchi by a Buddhist monk of the Silla Kingdom in Jangsusa Temple at Deokyusan Mountain [9]. These cases suggested that the tradition of making kimchi in jars could date as old as the Three Kingdoms period in Korea.

Indeed, the tradition of kimjang stems back from thousands of years ago, when it was originally a family ritual in autumn to ensure kimchi availability during winter. Then, the culture continued to somehow involve a broader audience, thus involving not only family members, but also the whole neighborhood, community or village. During the Joseon period, the tradition of preparing winter kimchi was well documented in different literature works. The first record of kimjang culture appears in the previously stated poem of Kyubo Lee entitled *Gapoyukyeong*, in which the scholar wrote: “radish pickle in soy sauce is good for three months in summer, salted cabbage for nine months of winter” [41]. Later on, many other Joseon scholars wrote about kimchi and kimjang culture in their literature works. Gwon Geun (1352–1409) in his essay collection *Yangchonjip* wrote: “It is October, the wind was rising and frost fell early in the morning / I have collected vegetables from the garden / Tasty winter kimchi has been prepared to get through the destitution of winter / It may not be a meal with delicacies, but every day I can have a tasty kimchi” [41]. Su-jung Kim (1624–1701) in *Gokwunjip* wrote: “Each household makes kimchi for winter / When autumn was deep, I harvested radish from the field” [41]. Yun-muk Park (1771–1849) in his poem collection *Jonjaejip* wrote: “How many rows of cabbage and radish in the field? / Are they enough to make winter kimchi to make it through three months of winter?” (extracted from the poem entitled *Seolhu*) and “When to do kimjang is hard to plan / As days pass, I get impatient” [38]. Other Joseon scholars mentioning kimchi and kimjang in their literature works include Geo-jeong Seo (1420–1488) in *Sakajip*, Chan-han Jo (1572–1631) in *Hyeonjujip*, Deok-mu Yi (1741–1793) in *Ajeong Yugo* and Gong-cheol Nam (1760–1840) in *Geumneungjip* [41].

## Modern history of kimchi

To arrive at the current point where kimchi is globally regarded as a healthy food, the journey has never been smooth and without any obstacle. Kimchi was once considered as an inferior food for the poor and despised for its strong odor [9]. However, with the studies demonstrating the health benefits of kimchi and the efforts of the Korean government to promote kimchi on the international stage through different international occasions, slowly but sure the value of kimchi could be improved until it became a precious food that represents the spirit of Korean people [10]. This chapter discusses how the commercialization and globalization of kimchi took place, the problems of kimchi recognition involving Korea and its neighboring countries (China and Japan), the challenges faced during the existence of kimchi and the establishment of kimchi image as a healthy food to attract global attention.

### Commercialization and globalization of kimchi

The commercialization and industrialization of kimchi production were done for the first time during South Korea’s involvement in the Vietnam war (1955–1975) [42]. The idea emerged as the Korean government wanted to provide rations for its troops. A request for help was then sent by the Korean government to the American government to ensure that South Korean troops, reportedly “desperate” for kimchi, could obtain it in the field. In the report of the South Korean Prime Minister (Il-kwon Chung) to the American President Lyndon B. Johnson in the White House in March 1967, the South Korean president Chung-hee Park expressed his concerns through a letter about the lack of kimchi that would hamper Korean participation in the Vietnam war [42]. He even expressed his personal experience during his military training in the USA that he had missed kimchi more than his own wife [43]. Therefore, the Korean government asked the American government to create a sustainable infrastructure to provide kimchi to the destitute Korean soldiers and support their morale. The program would cost three to four million dollars annually [43]. This event marked the first industrialization of kimchi. After the Vietnam war, kimchi was exported to the Middle East for Korean construction workers in the late 1970s and early 1980s [44].

True to its nature as a healthy food, kimchi made its way to the global stage through its first international debut at the worldwide sporting event 1984 Summer Olympic Games in Los Angeles, USA. It was introduced to foreigners for the first time and succeeded to capture the taste buds of the world. Later on, kimchi was designated as the official food for the 1986 Asian Games and 1988 Summer Olympic Games, both of which were held in Seoul, South Korea. These grand events provided a turning point for the recognition of kimchi at the international level and a sudden popularity as a world food [9]. Later on, the global image of kimchi became strongly associated with sport. These days, kimchi is regularly served to athletes at the athletes’ village cafeteria at international sporting competitions. In the 2012 Summer Olympic Games in London, Baron Sebastian Coe, the president of the London Olympic Games Committee, hosted three hundred distinguished guests at a banquet arranged with Korean dishes (including kimchi) in the Victoria & Albert Museum, and the foods received high praise [17]. At the 2014 Asian Games held in Incheon, South Korea, Un-Ju Kim, a North Korean female weightlifter who was a world record holder and a gold medalist at the event, said at the press conference that she regularly consumed kimchi as a healthy food and she did not need other special or expensive foods to support her health and performance [45]. In the 2010 FIFA World Cup in South Africa, South Korea made a big advertisement of a kimchi jar made in the shape of a soccer ball in order to promote kimchi [46].

The internationalization of K-food thrives as the influence of Korean wave (K-wave) through other sectors, such as fashion, beauty (cosmetics) and entertainment (films, dramas and songs) penetrates more countries and gains massive popularity, particularly among young generations. Many foreign people try K-food and kimchi after watching the actors eating them in their favorite Korean films or dramas. Many of them even flew to visit Korea to live a truly Korean gastronomic experience. In addition, the number of Korean restaurants is also increasing all over the world, including in metropolitan cities such as New York, London, and Paris, and the evaluation of gourmets of Korean dishes is changing in a positive manner. The internationalization and popularity of K-food and kimchi would certainly give a potential for gastro diplomacy [47].

A museum dedicated to kimchi named the Kimchi Field Museum (Kimchikan) was established in Seoul, South Korea in 1986. It was Korea’s first food museum. In 2000, the museum was renovated so as to expand and improve its facilities for visitors in anticipation of the third Asia-Europe Meeting in Seoul. The museum was reopened at Insa-dong, Jongno District, Seoul, in April 2015 [48]. In 2015, the museum was selected by the Cable News Network (CNN) as one of the world’s best food museums [49]. Its exhibits focus on the history of kimchi, its many historical and regional varieties, and its importance to Korean culture and cuisine.

To promote studies for further development of kimchi, the scientific and technological approach of kimchi has been emphasized with the proliferation of kimchi research centers and associations: Pulmuone, a kimchi producer, established its research team specialized in kimchi in 1985; Winia Mando, a South Korean household appliances manufacturer, created its research team in 1993; Hanyang Yutong, a chain of supermarkets, opened a research center in 1995 in Seoul; LG, a South Korean multinational electronics company, founded the Kimchi Research Center in 2022; and in the same year, Samsung, a kimchi refrigerator manufacturer, made an alliance with the Pulmuone research team [42]. The Kimchi Association of Korea was established in Busan, South Korea in August 2005 and is currently chaired by Ha-Yeon Lee, a renowned kimchi master [10, 22]. This association was founded to promote the globalization of kimchi and has set November 22 as South Korea’s national kimchi day [50]. Finally, in 2020, the World Institute of Kimchi, an affiliate institution of Korea Food Research Institute, was established in Gwangju, South Korea to promote research and development related to kimchi [51].

In 2008, South Korean scientists created a special “space kimchi” for So-yeon Yi, the first Korean astronaut, to take to space. Interestingly, the kimchi was bacteria-free, unlike normal kimchi that contains millions or billions of lactic acid bacteria (LAB) since they are essential for fermentation. It was feared that the cosmic ray might mutate the bacteria present in kimchi [52].

The Kimchi Bus Project was launched in 2011 by Si-hyeon Ryu, Young-dae Shim and Seok-bum Cho. The three South Koreans passionate about food traveled to 32 countries, starting in Russia and finishing in North America, with a primary goal of publicizing Korean food and culture to the world. In every country visited by the Kimchi Bus, the three acquaintances cooked traditional Korean food and spread knowledge about kimchi to international audience. The project was financially supported by the Korean government [53].

On February 7, 2013, the first lady of the USA Michelle Obama posted on her Twitter account that she harvested napa cabbage from the White House garden and prepared kimchi from it. She also shared her kimchi recipe in the post. This post has attracted the attention of international viewers and promoted kimchi toward an international audience [54].

The recognition of kimchi in the eye of the world culminated in 2013, when kimjang, the tradition of making and sharing kimchi in Korea was added to the list of UNESCO’s Representative List of Intangible Cultural Heritage of Humanity [36]. The demand for enlisting kimjang was submitted by both South Korea and North Korea, later inscribed in 2013 and 2015, respectively [55]. North Korean kimchi tends to be less spicy and red that South Korean kimchi due to less chili powder (*gochugaru*) used in the production. In addition, seafood is used less often and less salt is added in North Korean kimchi [56].

### Kimchi wars, disputes and other challenges

Kimchi as the symbol of Korean culture and identity has faced many challenges throughout its existence, particularly with regard to its origin and recognition as a Korean gastronomic heritage. The term “kimchi war” refers to a cultural dispute between Korea and its neighboring countries (China and Japan) regarding kimchi involving international organizations. This war has been related to many aspects in these countries, including politics, economics, and cultural identity [6, 10].

The “Korea-Japan kimchi standard disputes” in 1996 began with Korea protesting against Japanese commercial production of kimchi (namely *kimuchi*) arguing that such a product was different from kimchi [10]. In particular, Japanese *kimuchi* was not fermented and more similar to *asazuke*, a Japanese pickled vegetable characterized by short preparation time (30 min to several hours). Furthermore, Japan attempted to register its *kimuchi* as a Japanese original food at the Codex Alimentarius Commission held in Tokyo in 1996 while Korea also intended to register kimchi as a Korean original food at the same time [57]. The dispute drew wide media coverage. In 2001, the Codex Alimentarius published a voluntary standard defining kimchi as “a fermented food that uses salted napa cabbage as its main ingredient mixed with seasonings, and goes through a lactic acid production process at a low temperature” [58]. Following the inclusion of the kimchi standard, kimchi exports increased in Korea, but so did the production of kimchi in China and the import of Chinese kimchi into Korea [59].

In 2010, the price of kimchi rose greatly due to heavy rainfall shortening the harvesting time for cabbage and other main ingredients for kimchi. Korean and international media described such a phenomenon as a national crisis. Kimchi became scarce to find, expensive and unavailable in many Korean restaurants. In response to this crisis, the South Korean government reduced temporarily the tariffs on imported cabbage to coincide with the kimjang season [60].

The conflicts between Korea and China regarding kimchi arose several times between 2012 and 2020. Since 2012, the Chinese government has effectively banned the import of Korean kimchi to China by tightening the government regulations regarding the concentration of Bacillus present in pickled vegetable products (less than 30 colons per 100 g) [61]. Ignoring the standards of kimchi stipulated by the Codex Alimentarius in 2001, China defined kimchi as a derivative of its own *pao cai*. However, due to significantly different preparation techniques from *pao cai*, kimchi has significantly more lactic acid bacteria (LAB) through its fermentation process, which would exceed and never meet the Chinese standards for pickled vegetables. In 2017, Chinese media encouraged the boycott of Korean goods and Chinese nationalists vowed to not eat kimchi after South Korea accepted the deployment of Terminal High Altitude Area Defense (THAAD), an American anti-ballistic missile defense system on its land [62]. In November 2020, the International Organization for Standardization (ISO) posted new regulations for the production of *pao cai*. The same month, the Chinese news organization Global Times claimed the new ISO standard as an international standard for kimchi industries had been led by China. This sparked anger from South Korean media and people. Global Times then explained the controversy was due to a misunderstanding in translation [63]. The controversy was further exacerbated when the Chinese Ambassador of the United Nations Jun Zhang posted his photograph while holding up kimchi on his Twitter account as well as a Chinese YouTuber Ziqi Li with more than 14 million followers uploaded a kimchi-making video with the hashtag #ChineseFood [64]. This conflict resulted in the establishment of the new proper Chinese translation of Korean kimchi, *xin qi* (辛奇), to replace the previously used term *pao cai* that could lead to confusion with Chinese *pao cai* (泡菜) [65]. However, the word *xin qi* could be considered as a misleading expression because kimchi is not hot (辛) vegetables.

### Modern image of kimchi as a global healthy food

Korea is among the 10 countries in the world with the longest life expectancy (> 80 years) and this phenomenon is related to the high daily consumption of vegetables (*namul*) among Korean people, including kimchi [18, 66]. In 2030, a girl born in Korea is projected to live up to 90.8 years on average, thus being the highest life expectancy in any countries on earth [67]. Kimchi is globally renowned as a healthy food due to its abundance in beneficial compounds for health (nutrients and antioxidants) and low energy content (33.9 kcal/100 g *baechu* kimchi) [68]. In 2006, kimchi was reported to be one of the world’s healthiest foods by the American Journal of Public Health besides Indian lentil, Spanish olive oil, Greek yogurt and Japanese bean products [69]. Kimchi has been scientifically proven to exert nutritional and health-promoting properties, including antioxidative, antiaging, anticancer, antimicrobial, cholesterol-lowering, weight-controlling, and immune-stimulatory activities [70]. From the nutritional point of view, kimchi is an excellent source of fiber, vitamins, minerals and antioxidants [71]. The caveat of high kimchi consumption would be its relatively high concentration of sodium (781 mg/100 g) [68]. This amount corresponds to 52% of the adequate daily sodium intake (1500 mg) [72]. However, regular kimchi consumption was not associated with hypertension prevalence among Koreans [73]. In contrast, high kimchi intake (210 g/day) improved a better lipid blood profile associated with a lower risk of cardiovascular diseases in humans [74]. Scientists suggested that the overall healthy Korean diet composed of natural ingredients with low calorie intake would compensate for the high daily sodium intake and support the overall health of the Koreans [18].

Further development of kimchi would consist in exploring the functionalities of kimchi and promoting it as a functional food. Functional foods are foods that offer health benefits beyond their nutritional value [75]. This would be in line with the current image of kimchi as a healthy food. In addition to offering a balanced nutritional profile, kimchi is rich in lactic acid bacteria (LAB), the main actors behind the very fermentation of kimchi and complex flavor development throughout the process. The dominant LAB genera in kimchi are *Lactobacillus*, *Leuconostoc* and *Weissella*. Some of these LAB are generally known as probiotics, which are good beneficial bacteria intended to provide health benefits when consumed [76]. Fiber in kimchi (24% dry basis) can promote better blood sugar control and protect against conditions like diabetes, obesity, heart disease, stroke and digestive disorders [5, 71]. Kimchi also contains beneficial antioxidants that protect against premature aging and degenerative diseases, such as carotenoids, vitamin C, flavonoids and other phenolic compounds originally found in the natural ingredients (also known as phytochemicals) [71].

During the difficult times of severe acute respiratory syndrome (SARS) outbreak in South China and its neighboring countries in 2003, Korea only recorded three cases of infection with no death while the cases in China, Hong Kong and Taiwan reached approximately 7,500 cases and 720 deaths [77]. The resistance of Korean people against SARS infection was associated with regular kimchi consumption as a healthy food [78].

In the times of global COVID-19 (SARS-CoV-2) pandemic commencing in late 2019, high consumption of fermented vegetables has been shown to correlate with lower mortality rates in East Asia (including Korea), Central Europe and the Balkans [79]. Specifically, kimchi was reported to improve cellular antioxidant status by activating the nuclear factor (erythroid-derived 2)-like 2 (Nrf2)-related pathway which would be helpful in mitigating COVID-19 severity [80]. In addition, kimchi was also demonstrated to inhibit the production of angiotensin-converting enzyme 2 (ACE 2), which is the binding site for coronavirus, thus preventing COVID-19 [81]. Furthermore, probiotics in kimchi may prevent intestinal microbiota dysbiosis and help establish a healthy gut with a balanced microbial composition that would in turn support the management of respiratory tract viral infections through the gut-lung axis [82]. Some LAB isolated from kimchi have also been shown to possess immunomodulatory effects that would strengthen the immune system against COVID-19 [83]. However, further studies and more scientific data seem to be needed to prove the beneficial effects of kimchi related to COVID-19.

## The face of kimchi today

Today, kimchi is known at a global level as a healthy food from Korea. The national industrialization in Korea beginning in the 1960s has at some point participated in the modernization of kimchi. The invention of kimchi fridge in the 1980s has made kimchi preparation much easier and it was indeed a cultural hallmark in the history of kimchi [84]. Korean people no longer needed to prepare kimchi and keep it in big jars outside their houses. A kimchi fridge is able to control the temperature for a slow and optimal fermentation of kimchi. Modern agriculture, along with kimchi fridge also allows to ensure the availability of all types of kimchi throughout the year, independently of the seasons. Today, every Korean family possesses at least a kimchi fridge in their house [41]. With the industrialization, many Korean people left their small hometowns and moved to big cities where they lived in smaller apartments with fewer family members. Some of them missed kimjang while others made an effort to come back home in autumn to participate in kimjang in their respective hometowns with their families and communities. Despite the current modernization, kimjang is predicted to last in Korean culture and will surely continue adjusting to social changes [38, 40].

Nowadays, kimchi has been widely industrialized and is practically always available at the market all year long [2]. The annual size of kimchi market in Korea reached USD 1.2 million in 2017 while at the global scale, the growth potential of global kimchi market attained USD 2.39 billion as of 2020–2024 [22]. In 2018, Japan was the main importer of Korean kimchi (65,373 tons), followed by the USA (10,280 tons), Thailand (6,347 tons), Hong Kong (5,121 tons) and Australia (3,745 tons) [22].

**Fig. 4:**
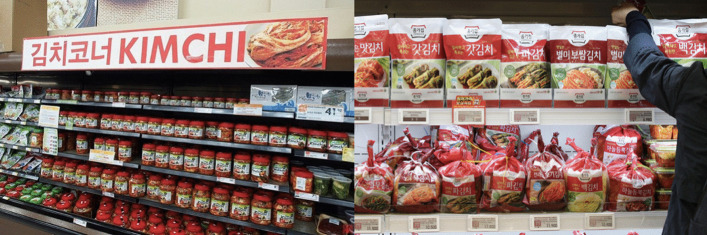
Packaged ready-to-eat kimchi products widely displayed and sold in Korean supermarkets in France (left) and South Korea (right)

For practical reasons, packaged kimchi (Fig. 4) is ubiquitous in Korean market and often opted instead of creating kimchi from vegetables. All types of kimchi are available in packaging that can be easily chosen in the market and directly consumed without any further required preparation. Commercial kimchi is usually packaged in glass jars or plastic pouches. Currently, the three basic packages of kimchi products found in the market include: (1) freshly packed unfermented salad-type kimchi (called *geotjeori* or fresh kimchi, seasoned, without fermentation), (2) refrigerated fermented kimchi (which still contains bacteria and continues to ferment during storage) and (3) pasteurized or sterilized fermented kimchi (which has the longest shelf life compared to the previous two types of kimchi and contains little or no microorganisms due to the application of heating) [2]. The sales of packaged kimchi have demonstrated a significant increase from 540,000 tons in 2010 to 710,000 tons in 2017 [22]. In contrast, even though more Koreans still prefer to prepare homemade kimchi than to buy packaged kimchi, the number of homemade kimchi consumption decreased from 1,460,000 tons in 2010 to 1,220,000 tons in 2017 [22].

**Fig. 5:**
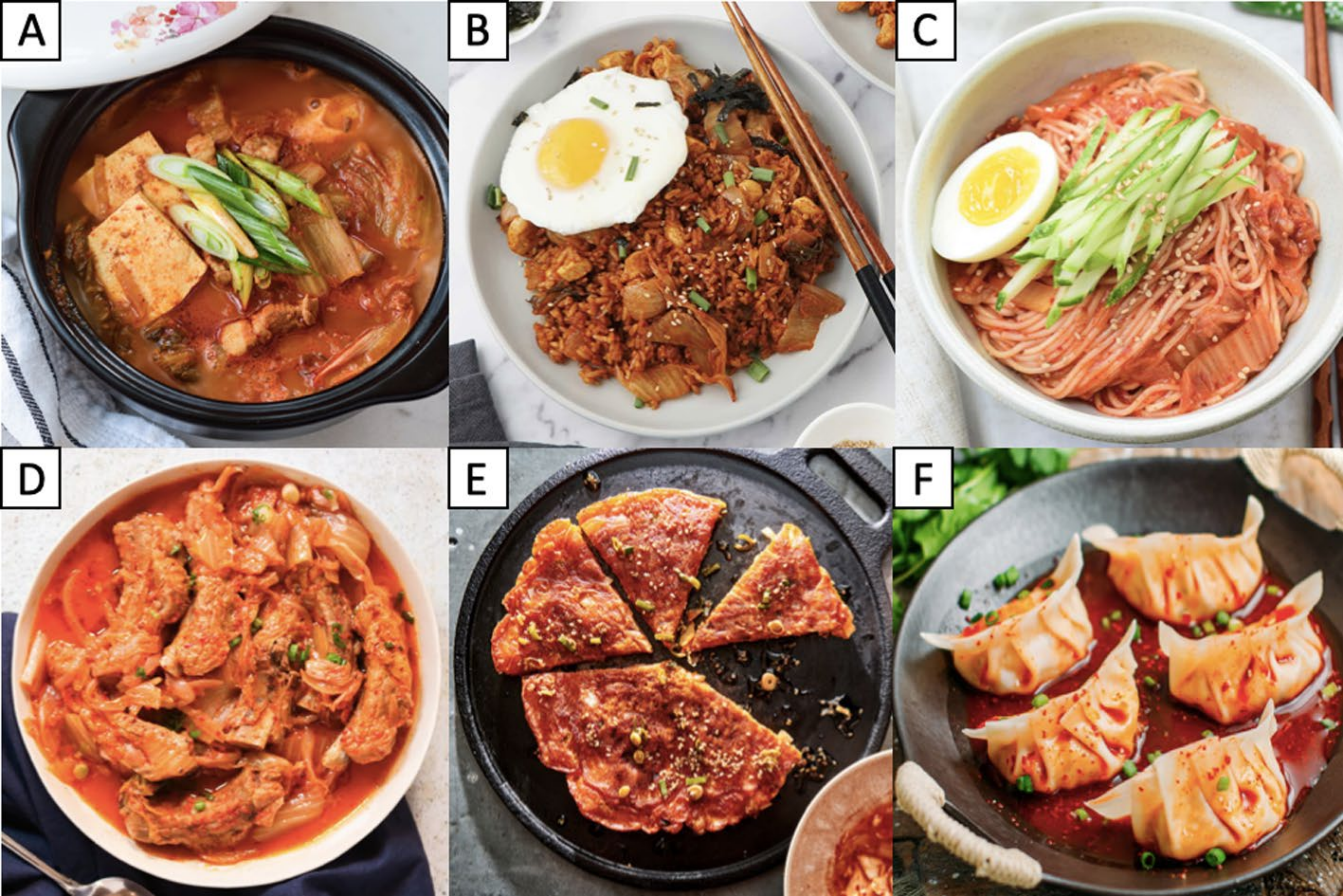
Different traditional Korean dishes made using kimchi, including **A** kimchi stew (*kimchi jjigae*), **B** kimchi fried rice (*kimchi bokkeumbap*), **C** kimchi noodles (*kimchimari guksu*), **D** braised kimchi (*kimchi jjim*), **E** kimchi pancake (*kimchi jeon*) and **F** kimchi dumplings (*kimchi mandu*)

**Fig. 6:**
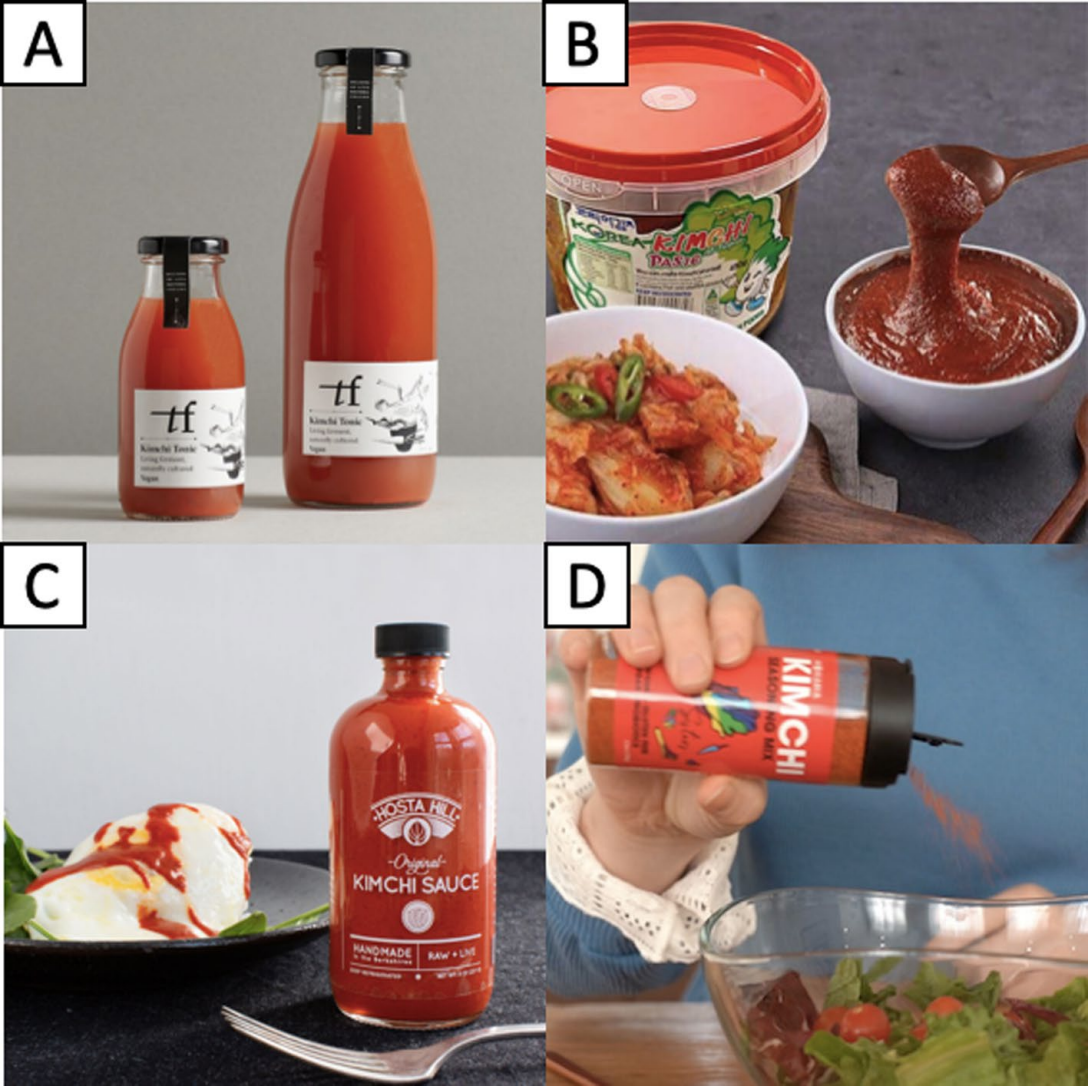
Some popular and emerging international food products derived from kimchi, including **A** kimchi juice, **B** kimchi paste, **C** kimchi sauce and **D** kimchi seasoning powder

Nowadays, there are about 200 variations of kimchi existing in Korea that vary according to different main ingredients used, regions of origin and seasons [2]. In addition, today’s kimchi is no longer merely consumed as a side dish. Some Korean dishes incorporate kimchi and highlight kimchi as their main ingredients, such as kimchi stew (*kimchi jjigae*), kimchi fried rice (*kimchi bokkeumbap*), kimchi noodles (*kimchimari guksu*), braised kimchi (*kimchi jjim*), kimchi pancake (*kimchi jeon*) and kimchi dumplings (*kimchi mandu*) (Fig. 5) [53]. The international trend of consuming kimchi has given birth to the creation of food products derived from kimchi, including kimchi juice, kimchi paste, kimchi sauce and kimchi seasoning powder (Fig. 6) [22]. In many countries, kimchi has undergone acculturation and has been incorporated into foods originating from other countries, such as pizzas, sandwiches, burgers, tacos and burritos [9, 22].

## Kimchi polemics

Throughout its existence for millennia on Korean land, the origin and identity of kimchi have been challenged by some polemics and disagreements questioning its originality as the national food of Korea. The article written by Jang et al*.* in 2015 [6] addressed specifically the polemics regarding the origin of kimchi previously published by Joo in 1994 and 2010 [85, 86].

**Fig. 7:**
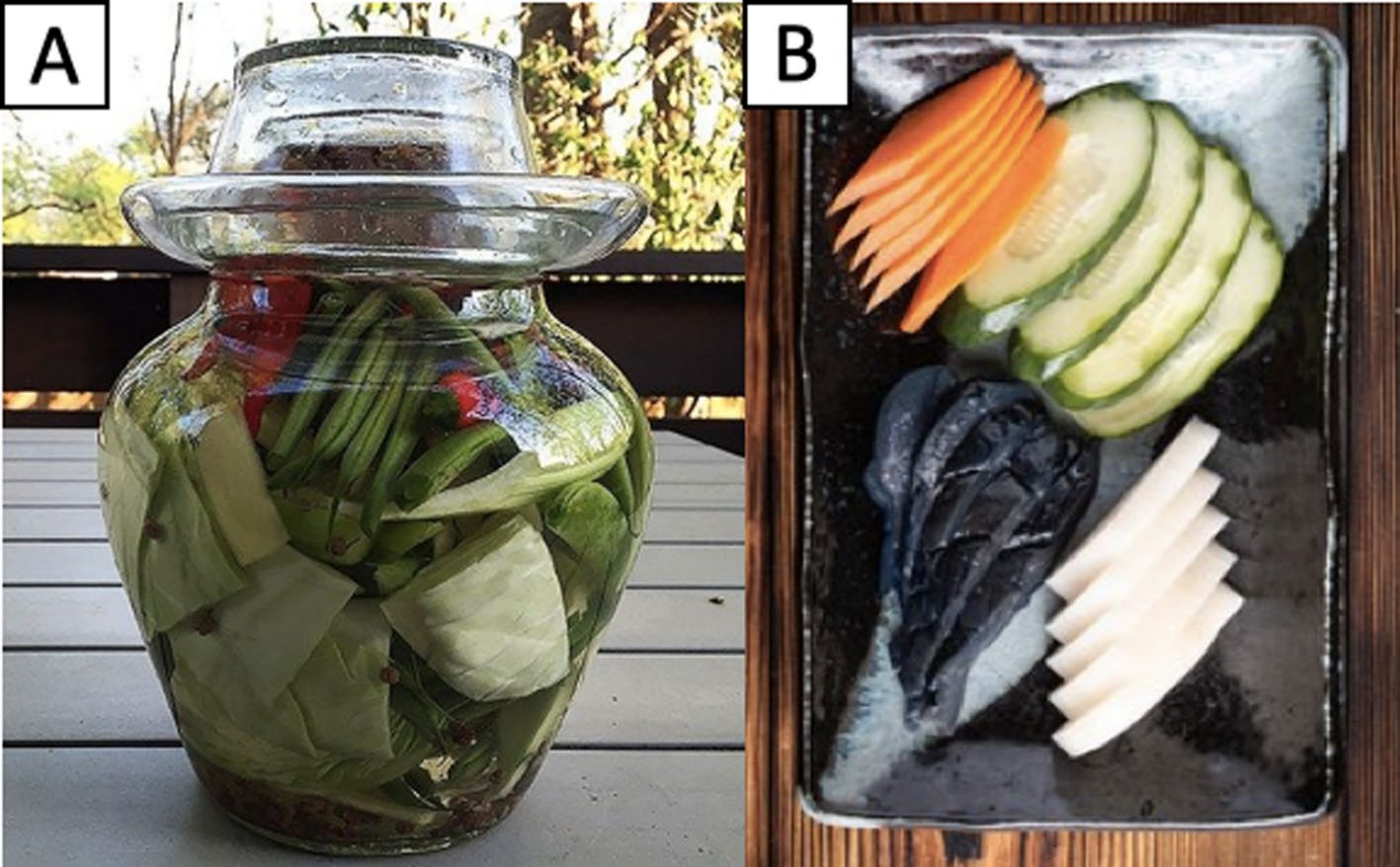
**A** Chinese *pao cai* and **B** Japanese *tsukemono*, two fermented vegetable foods from Asia that have been constantly compared to kimchi with regard to their roots and originality. However, these are completely different foods from kimchi. Unlike kimchi that is a fermented food, *pao cai* and *tsukemono* are not made through fermentation

Firstly, kimchi was said to have the same root as Chinese *pao cai* (Fig. 7A) and Japanese *tsukemono* (Fig. 7B), both of which are made from vegetables. However, the principle of kimchi production is different from *pao cai* and *tsukemono*. While the making of *pao cai* and *tsukemono* consists of the addition of salt to reduce water activity and vinegar to prevent microbial activities by reducing pH, the production of kimchi relies on fermentation that encourages the growth of microorganisms. The word *jeo* (菹) as appeared in *Sikyung* was argued to refer to *pao cai*. However, the word was widely used in many Korean literature works later to describe kimchi. Therefore, kimchi can be said to have an original root referring to the Korean culture that differs from *pao cai* or *tsukemono* [6].

Secondly, the kimchi as we know today (red in color due to the use of chili powder) was said to be invented only in 1592 at the time of Imjin wars (Japanese invasion of Korea), during which red chili pepper was first introduced in Korea. Prior to this event, kimchi was white and made without chili pepper. *Gochu* (Korean red chili pepper), a special cultivar of *Capsicum annuum* commonly used to season cabbages in kimchi making, has existed in the Korean peninsula since 2,000 years ago according to Korean official documents *Samguk Sagi* and *Mankiyoram* [6]. In addition, *gochu* has been shown to be present in the Korean peninsula since billions of years ago and it is indeed native to Korea [11, 35]. Biologically, Korean *gochu* is also different from the red chili peppers from other Asian and Central American countries that would be too spicy to be applied in kimchi [11]. According to these facts, chili pepper has been used in kimchi production since thousands of years ago [6].

Finally, kimchi was said to be originally made of white radish and cabbage kimchi was only developed in the nineteenth century. It was also said that napa cabbage was imported to Korea from China during the Goryeo period and it took several centuries to adapt the cultivar to grow in Joseon. However, *Samguk Sagi* clearly demonstrates the existence of cabbage kimchi in Korea 1500 years ago through a proverb “I want to tear a person limb from limb like the way we tear kimchi.” From such a description of tearing kimchi, one may conclude that the kimchi referred to in this document is cabbage kimchi [6]. Moreover, the existence of cabbage in Korea can be traced back from as early as the thirteenth century in many classical literature works containing the word *sung* (菘) referring to cabbage, including the *Hunmongjahoe* by Choi (1527), the *Dongkuk-isangkukjip* by Lee (1241), the *Dongmunseon* by Seo (1478), the *Sakajip* by Seo (1488) and the *Jeompiljae* by Kim (1497) [6]. Other literature works from the sixteenth and seventeenth centuries also incorporate the words *sungchae* (菘菜) and *baekchae* (白菜) that also represent cabbage [6]. Based on these facts, cabbage has existed for thousands of years in Korea and been used in kimchi making [6].

## Conclusions

Kimchi has existed and been an integral part of the Korean culture for thousands of years. Numerous classical literature works allow to trace the presence of kimchi as early as 3000 years ago. Today, kimchi is globally known as a healthy food from Korea. During the time travel for millennia, kimchi has faced many challenges that put its identity as an original Korean dish into question, particularly with Japan and China as Korea’s neighboring countries. Such cultural disputes are often addressed as “kimchi wars” that have attracted the attention of international media. The standardization of kimchi by the Codex Alimentarius Commission in 2001 and the recognition of kimjang as a UNESCO’s Intangible Cultural Heritage of Humanity in 2013 were the hallmark cultural events that reinforced the sovereignty of kimchi at the international level. Currently, kimchi still thrives along with the Korean wave (K-wave) that is gaining popularity in many countries through Korean influence in international entertainment industry, particularly music and drama. Kimchi is used as a soft power for gastro diplomacy to introduce the Korean culture and build the brand image of Korea to the world.

## Data Availability

All the literature works that have been used in the writing of this manuscript are available in the references section.

## References

[1] Meigs A, Counihan C, Van Esterik P (1997). Food as a cultural construction. Food and culture: a reader.

[2] Hongu N, Kim AS, Suzuki A, Wilson H, Tsui KC, Park S (2017). Korean kimchi: promoting healthy meals through cultural tradition. J Ethn Foods.

[3] Park KY, Cheigh HS, Hui YH, Meunier-Goddik L, Hansen AS, Josephsen J, Nip WK, Stanfield PS, Toldrá F (2004). Kimchi. Handbook of food and beverage fermentation technology.

[4] Surya R, Lee AGY (2022). Exploring the philosophical values of kimchi and kimjang culture. J Ethn Foods.

[5] Cheigh HS, Park KY, Lee CY (1994). Biochemical, microbiological, and nutritional aspects of kimchi (Korean fermented vegetable products). Crit Rev Food Sci Nutr.

[6] Jang DJ, Chung KR, Yang HJ, Kim KS, Kwon DY (2015). Discussion on the origin of kimchi, representative of Korean unique fermented vegetables. J Ethn Foods.

[7] Jung JY, Lee SH, Jeon CO (2014). Kimchi microflora: history, current status, and perspectives for industrial kimchi production. Appl Microbiol Biotechnol.

[8] Yoon D. Development of kimchi and income growth. In: The humanistic understanding of kimchi. Gwangju: World Institute of Kimchi; 2015.

[9] Lim J. Acknowledgement of kimchi’s value to humanity and the globalization of kimchi. In: The humanistic understanding of kimchi. Gwangju: World Institute of Kimchi; 2015.

[10] Cho HS (2006). Food and nationalism. Korean J Int Stud.

[11] Kwon DY, Jang DJ, Yang HJ, Chung KR (2014). History of Korean *gochu*, *gochujang*, and kimchi. J Ethn Foods.

[12] Kwon DY, Shin DH, Shetty K (2022). The answer for a healthy life is a Korean traditional diet. Korean food systems: secrets of the K-diet for healthy aging.

[13] Oh SH, Park KW, Daily JW, Lee YE (2014). Preserving the legacy of healthy Korean food. J Med Food.

[14] Park EY (2022). Korea: a history.

[15] Kwon DY, Chung KR, Jang DJ (2019). The history and science of chongkukjang, a Korean fermented soybean product. J Ethn Foods.

[16] Lee GA (2011). The transition from foraging to farming in prehistoric Korea. Curr Anthropol.

[17] Oktay S, Ekinci EK (2019). Medicinal food understanding in Korean gastronomic culture. J Ethn Foods.

[18] Kim SH, Kwon DY, Shin D (2020). Namul, the driving force behind health and high vegetable consumption in Korea. J Ethn Foods.

[19] Shoda S, Lucquin A, Ahn JH, Hwang CJ, Craig OE (2017). Pottery use by early Holocene hunter-gatherers of the Korean peninsula closely linked with the exploitation of marine resources. Quat Sci Rev.

[20] Crawford GW, Lee GA (2003). Agricultural origins in the Korean peninsula. Antiquity.

[21] Pettid MJ (2008). Korean cuisine: an illustrated history.

[22] Park C, Lee E, editors. Magazine F: kimchi. Seoul: Woowa Brothers, Magazine B; 2020.

[23] Park J, Lee HJ (2017). Shifts in kimchi consumption between 2005 and 2015 by region and income level in the Korean population: Korea National Health and Nutrition Examination Survey (2005, 2015). Korean J Community Nutr.

[24] Kim SH, Kim MS, Lee MS, Park YS, Lee HJ, Kang SA, Lee HS, Lee KE, Yang HJ, Kim MJ, Lee YE, Kwon DY (2016). Korean diet: characteristics and historical background. J Ethn Foods.

[25] Cawley KN (2019). Religious and philosophical traditions of Korea.

[26] Yoo T, Yoon I-J (2015). Becoming a vegetarian in Korea: the sociocultural implications of vegetarian diets in Korean society. Korea J.

[27] Kim J, Shim J-M, Kim S (2022). Confucian identification, ancestral beliefs, and ancestral rituals in Korea. Religions.

[28] Chung HK, Yang HJ, Shin D, Chung KR (2016). Aesthetics of Korean foods: the symbol of Korean culture. J Ethn Foods.

[29] Sung KT (1995). Measures and dimensions of filial piety in Korea. Gerontologist.

[30] Di Schino J, Saberi H (2011). Kimchi: ferment at the heart of Korean cuisine, from local identity to global consumption. Cured, fermented, and smoked foods.

[31] Zhang J (2015). The food of the worlds: mapping and comparing contemporary gastrodiplomacy campaigns. Int J Commun.

[32] Deniar SM, Effendi TD (2019). Halal food diplomacy in Japan and South Korea. J Soc Polit Sci.

[33] Shin B (2013). Emerging issues leading Korea into a dream society. J Futures Stud.

[34] Pae HK, Pae HK (2018). The Korean writing system, Hangul and word processing. Writing systems, reading processes and cross-linguistic influences: reflections from the Chinese, Japanese and Korean languages.

[35] Yang HJ, Chung KR, Kwon DY (2017). DNA sequence analysis tells the truth of the origin, propagation, and evolution of chili (red pepper). J Ethn Foods.

[36] Lee CH, Ahn BS (1995). Literature review on kimchi, Korean fermented vegetable foods I. history of kimchi making. J Korean Soc Food Cult..

[37] Kang J. Korean food culture and the making of winter kimchi, kimjang. In: The humanistic understanding of kimchi. Gwangju: World Institute of Kimchi; 2015.

[38] Hwang G. Challenges and the prospect for the sustainable protection of the “kimjang culture”, a UNESCO Intangible Cultural Heritage of Humanity. In: The humanistic understanding of kimchi. Gwangju: World Institute of Kimchi; 2015.

[39] Alper T. Korean kimchi’s secret ingredient: a taste of community spirit. 2021. Available from: https://www.korea.net/NewsFocus/Opinion/view?articleId=194453. Accessed 17 August 2022.

[40] Kang J. Modernization and continuation of kimjang culture. In: The humanistic understanding of kimchi and kimjang culture. Gwangju: World Institute of Kimchi; 2014.

[41] Park C. Korean kimjang culture: history, significance and culture. In: The humanistic understanding of kimchi and kimjang culture. Gwangju: World Institute of Kimchi; 2014.

[42] Jo JS (2016). History of kimchi industry. Food Sci Ind.

[43] Karp M. How kimchi was used as political leverage in the Vietnam war. 2018. Available from: https://www.vice.com/en/article/bjpdxd/how-kimchi-was-used-as-political-leverage-in-the-vietnam-war. Accessed 3 September 2022.

[44] Han KK, Kendall L (2010). The “kimchi wars” in globalizing East Asia: consuming class, gender, health and national identity. Consuming Korean tradition in early and late modernity.

[45] Baek BY. Kimchi helped me win the gold. 2014. Available from: http://www.koreatimes.co.kr/www/nation/2017/05/207_165370.html. Accessed 11 September 2022.

[46] Jiao P. Kimchi ad kicks off storm in a Sichuan pot. 2010. Available from: https://www.scmp.com/article/717417/kimchi-ad-kicks-storm-sichuan-pot. Accessed 11 September 2022.

[47] Nye J, Kim Y, Kim Y (2019). Soft power and the Korean wave. South Korean popular culture and North Korea.

[48] Cho H (2013). Fermentation of intangible cultural heritage: interpretation of kimchi in museums. Mus Manag Curatorship.

[49] Hinson T. 11 of the world’s top food museums. 2015. Available from: https://edition.cnn.com/travel/article/worlds-top-food-museums/index.html#:~:text=Kimchi%20Museum%20(Seoul),papers%20about%20the%20pickled%20product. Accessed 6 September 2022.

[50] Park R. California officially designates Nov. 22 as kimchi day. 2021. Available from: http://tbs.seoul.kr/eFm/newsView.do?typ_800=I&idx_800=3447065&seq_800=. Accessed 8 September 2022.

[51] Kim EY (2013). World Institute of Kimchi as a leading global institute of fermented foods. Biotechnol J.

[52] Choe SH. Kimchi goes to space, along with first Korean astronaut. 2008. Available from: https://www.nytimes.com/2008/02/22/world/asia/22iht-kimchi.1.10302283.html. Accessed 13 September 2022.

[53] Ramirez E. Last stop pn the kimchi bus. 2014. Available from: https://www.koreaherald.com/view.php?ud=20140817000330. Accessed 13 September 2022.

[54] Bahk EJ. Michelle Obama is a lover of kimchi. 2013. Available from: http://www.koreatimes.co.kr/www/news/nation/2013/02/116_130259.html. Accessed 13 September 2022.

[55] Cwiertka KJ, King MT (2019). From military rations to UNESCO Heritage: a short history of Korean kimchi. Culinary nationalism in Asia.

[56] Lee CHJ (2005). Eating Korean: from barbecue to kimchi, recipes from my home.

[57] Sims C. Cabbage is cabbage? Not to kimchi lovers; Koreans take issue with a rendition of their national dish made in Japan. 2000. Available from: https://www.nytimes.com/2000/02/05/business/cabbage-cabbage-not-kimchi-lovers-koreans-take-issue-with-rendition-their.html. Accessed 13 September 2022.

[58] Codex Alimentarius International Food Standards. Standard for kimchi CXS 223–2001. 2017. Available from: https://www.fao.org/fao-who-codexalimentarius/sh-proxy/es/?lnk=1&url=https%253A%252F%252Fworkspace.fao.org%252Fsites%252Fcodex%252FStandards%252FCXS%2B223-2001%252FCXS_223e.pdf. Accessed 13 September 2022.

[59] King MT, King MT (2019). Introduction: Culinary nationalism in Asia. Culinary nationalism in Asia.

[60] Glionna JM. Kimchi crisis leaves South Koreans hot under collar. 2010. Available from: https://www.latimes.com/archives/la-xpm-2010-oct-05-la-fg-south-korea-kimchi-20101005-story.html. Accessed 13 September 2022.

[61] Chung JW. Korea resumes kimchi export to China. 2015. Available from: https://www.koreaherald.com/view.php?ud=20151224000976. Accessed 13 September 2022.

[62] Hernandez JC. South Korean stores feel China’s wrath as U.S. missile system is deployed. 2017. Available from: https://www.nytimes.com/2017/03/09/world/asia/china-lotte-thaad-south-korea.html. Accessed 13 September 2022.

[63] Kim D, Mah S. South Koreans, Chinese clash on social media over Chinese-style kimchi winning international certificate. 2020. Available from: https://www.reuters.com/article/us-southkorea-china-kimchi-idUSKBN28A2NQ. Accessed 13 September 2022.

[64] Kang HM. Korea and China clash over kimchi origin following post by Chinese ambassador. 2021. Available from: http://koreabizwire.com/korea-and-china-clash-over-kimchi-origin-following-post-by-chinese-ambassador/179534. Accessed 13 September 2022.

[65] Wong MH. Kimchi’s new Chinese name has become the epicenter of a cultural war… again. 2021. Available from: https://edition.cnn.com/travel/article/xinqi-kimchi-new-chinese-name-cmd/index.html. Accessed 13 September 2022.

[66] United Nations Development Programme. Human development report 2020. 2020. Available from: https://hdr.undp.org/sites/default/files/hdr2020.pdf. Accessed 13 September 2022.

[67] Gallagher J. Life expectancy to break 90 barrier by 2030. Available from: https://www.bbc.com/news/health-39040146. Accessed 13 September 2022.

[68] U.S. Department of Agriculture. Kimchi. 2020. Available from: https://fdc.nal.usda.gov/fdc-app.html#/food-details/1103667/nutrients. Accessed 13 September 2022.

[69] Raymond J. World’s heathiest foods: kimchi (Korea). 2013. Available from: https://www.health.com/condition/digestive-health/worlds-healthiest-foods-kimchi-korea. Accessed 13 September 2022.

[70] Chang HC (2018). Healthy and safe Korean traditional fermented foods: kimchi and chongkukjang. J Ethn Foods.

[71] Park KY, Ju J, Park KY, Kwon DY, Lee KW, Park S (2018). Kimchi and its health benefits. Korean functional foods: composition, processing, and health benefits.

[72] World Health Organization. Salt reduction. 2020. Available from: https://www.who.int/news-room/fact-sheets/detail/salt-reduction. Accessed 13 September 2022.

[73] Song HJ, Lee HJ (2014). Consumption of kimchi, a salt fermented vegetable, is not associated with hypertension prevalence. J Ethn Foods.

[74] Choi IH, Noh JS, Han JS, Kim HJ, Han ES, Song YO (2013). Kimchi, a fermented vegetable, improves serum lipid profiles in healthy young adults: a randomized clinical trial. J Med Food.

[75] Khan RS, Grigor J, Winger R, Win A (2013). Functional food product developmentZ—opportunities and challenges for food manufacturers. Trends Food Sci Technol.

[76] Park KY, Jeong J, Lee YE, Daily JW (2014). Health benefits of kimchi (Korean fermented vegetables) as a probiotic food. J Med Food.

[77] World Health Organization. Summary of probable SARS cases with onset of illness from 1 November 2002 to 31 July 2003. 2015. Available from: https://www.who.int/publications/m/item/summary-of-probable-sars-cases-with-onset-of-illness-from-1-november-2002-to-31-july-2003. Accessed 13 September 2022.

[78] Chazan D. Korean dish ‘may cure bird flu’. 2005. Available from: http://news.bbc.co.uk/2/hi/asia-pacific/4347443.stm. Accessed 13 September 2022.

[79] Das G, Heredia JB, de Lourdes PM, Coy-Barrera E, Oliveira SMR, Guttierez-Grijalva EP, Cabanillas-Bojorquez LA, Shin HS, Patra JK (2021). Korean traditional foods as antiviral and respiratory disease prevention and treatments: a detailed review. Trends Food Sci Technol.

[80] Bousquet J, Anto JM, Czarlewski W, Haahtela T, Fonseca SC, Iaccarino G, Blain H, Vidal A, Sheikh A, Akdis CA, Zuberbier T, ARIA group. Cabbage and fermented vegetables: from death rate heterogeneity in countries to candidates for mitigation strategies of severe COVID-19. Allergy. 2021;76(3):735–750. 10.1111/all.14549 PMC7436771 32762135

[81] Bousquet J, Czarlewski W, Zuberbier T, Mullol J, Blain H, Cristol JP, De La Torre R, Pizarro Lozano N, Le Moing V, Bedbrook A, Agache I, Akdis CA, Canonica GW, Cruz AA, Fiocchi A, Fonseca JA, Fonseca S, Gemicioglu B, Haahtela T, Iaccarino G, Ivancevich JC, Jutel M, Klimek L, Kraxner H, Kuna P, Larenas-Linnemann DE, Martineau A, Melen E, Okamoto Y, Papadopoulos NG, Pfaar O, Regateiro FS, Reynes J, Rolland Y, Rouadi PW, Samolinski B, Sheikh A, Toppila-Salmi S, Valiulis A, Choi HJ, Kim HJ, Anto JM (2021). Potential interplay between Nrf2, TRPA1, and TRPV1 in nutrients for the control of COVID-19. Int Arch Allergy Immunol.

[82] Bottari B, Castellone V, Neviani E (2021). Probiotics and Covid-19. Int J Food Sci Nutr.

[83] Singh K, Rao A (2021). Probiotics; a potential immunomodulatory in COVID-19 infection management. Nutr Res.

[84] Moon SH, Kim EJ, Kim EJ, Chang HC (2018). Development of fermentation: storage mode for kimchi refrigerator to maintain the best quality of kimchi during storage. Korean J Food Sci Technol.

[85] Joo YH (1994). Kimchi, Korean ethno-foods: anthropology of kimchi.

[86] Joo YH (2010). Food war and culture war.

[87] Hoa Q. Kimjang culture of making and sharing kimchi. Available from: https://vovworld.vn/en-US/cultural-rendezvous/kimjang-culture-of-making-and-sharing-kimchi-471459.vov. Accessed 17 October 2022.

